# Two Novel Alkaliphilic Species Isolated from Saline-Alkali Soil in China: *Halalkalibacter flavus* sp. nov., and *Halalkalibacter lacteus* sp. nov

**DOI:** 10.3390/microorganisms12050950

**Published:** 2024-05-08

**Authors:** Pin-Jiao Jin, Lei Sun, Yong-Hong Liu, Kang-Kang Wang, Manik Prabhu Narsing Rao, Osama Abdalla Abdelshafy Mohamad, Bao-Zhu Fang, Li Li, Lei Gao, Wen-Jun Li, Shuang Wang

**Affiliations:** 1 Heilongjiang Academy of Black Soil Conservation and Utilization, Postdoctoral Station of Heilongjiang Academy of Agricultural Sciences, Harbin 150086, China; jinpinjiao1992@163.com (P.-J.J.); tufeisuosunlei@163.com (L.S.); wk137xc@163.com (K.-K.W.); 2 State Key Laboratory of Desert and Oasis Ecology, Key Laboratory of Ecological Safety and Sustainable Development in Arid Lands, Xinjiang Institute of Ecology and Geography, Chinese Academy of Sciences, Urumqi 830011, China; liuyonghong18@163.com (Y.-H.L.); osama@aru.edu.eg (O.A.A.M.); fangbaozhu@ms.xjb.ac.cn (B.-Z.F.); lili.bobo@outlook.com (L.L.); gaolei19@mails.ucas.ac.cn (L.G.); 3 Instituto de Ciencias Aplicadas, Facultad de Ingeniería, Universidad Autónoma de Chile, Sede Talca, Talca 3460000, Chile; deene.manik@gmail.com; 4 Xinjiang Key Laboratory of Biodiversity Conservation and Application in Arid Lands, Xinjiang Institute of Ecology and Geography, Chinese Academy of Sciences, Urumqi 830011, China; 5 State Key Laboratory of Biocontrol and Guangdong Key Laboratory of Plant Resources, School of Life Sciences, Sun Yat-Sen University, Guangzhou 510275, China

**Keywords:** *Halalkalibacter*, polyphasic taxonomy, saline-alkaline soil, two novel species

## Abstract

The degradation of farmland in China underscores the need for developing and utilizing saline-alkali soil. Soil health relies on microbial activity, which aids in the restoration of the land’s ecosystem, and hence it is important to understand microbial diversity. In the present study, two Gram-stain-positive strains HR 1-10^T^ and J-A-003^T^ were isolated from saline-alkali soil. Preliminary analysis suggested that these strains could be a novel species. Therefore, the taxonomic positions of these strains were evaluated using polyphasic analysis. Phylogenetic and 16S rRNA gene sequence analysis indicated that these strains should be assigned to the genus *Halalkalibacter*. Cell wall contained *meso*-2,6-diaminopimelic acid. The polar lipids present in both strains were diphosphatidyl-glycerol, phosphatidylglycerol, and an unidentified phospholipid. The major fatty acids (>10%) were anteiso-C_15:0_, C_16:0_ and iso-C_15:0_. Average nucleotide identity and digital DNA#x2013;DNA hybridization values were below the threshold values (95% and 70%, respectively) for species delineation. Based on the above results, the strains represent two novel species of the genus *Halalkalibacter*, for which the names *Halalkalibacter flavus* sp. nov., and *Halalkalibacter lacteus* sp. nov., are proposed. The type strains are HR 1-10^T^ (=GDMCC 1.2946^T^ = MCCC 1K08312^T^ = JCM 36285^T^), and J-A-003^T^ (=GDMCC 1.2949^T^ = MCCC 1K08417^T^ = JCM 36286^T^).

## 1. Introduction

Songnen Plain is the second largest plain in China (42°30′~51°20′ N, 121°40′~128°30′ E) and it serves as a significant agricultural and animal husbandry center in the northern region, known for its extensive distribution of saline-alkali soil [1]. Songnen Plain was previously one of China’s three main grasslands and is currently among the top three regions globally, with the highest concentration of sodic-saline soil [2]. These saline-alkali soils are mainly concentrated in the western part of Jilin and Heilongjiang provinces, and their permeability to fresh water is slow due to the high content of montmorillonite and sodium bicarbonate [3].

Soil salinization poses a significant environmental challenge. Investigation of soil properties has revealed that the salinization of grasslands profoundly affects the physical, chemical, and biological aspects of soil, along with the metabolic diversity of soil bacteria [4], since soil health relies on microbial activity and the salinity levels in grasslands are closely linked to the composition and functioning of soil microorganisms [5]. In saline-alkali soil, salt and alkali frequently coexist, presenting a dual stress, and as a response, numerous haloalkaliphiles have emerged, with distinctive structures and physiological adaptations, to thrive in such conditions [2,6]. In the past, both culture-dependent and independent microbial diversity analysis were carried out to understand the microbial community structure [2,7,8], and many novel halophilic and alkaliphilic species were also described from this area [8,9,10,11,12]. During microbial diversity analysis of such an ecosystem, strains HR 1-10^T^ and J-A-003^T^ were isolated. The preliminary characterization (based on 16S rRNA gene sequence analysis) showed that these strains belong to the genus *Halalkalibacter,* but could be novel at the species level, and hence these strains were selected for further studies.

The genus *Halalkalibacter* was proposed by Joshi et al. [13]. Currently, the genus *Halalkalibacter* consists of 10 validly published species names (*Halalkalibacter akibai*, *Halalkalibacter alkalisediminis*, *Halalkalibacter hemicellulosilyticus*, *Halalkalibacter kiskunsagensis*, *Halalkalibacter krulwichiae*, *Halalkalibacter nanhaiisediminis*, *Halalkalibacter oceani*, *Halalkalibacter okhensis*, *Halalkalibacter urbisdiaboli*, and *Halalkalibacter wakoensis*, and “*Halalkalibacter alkaliphilus*” not validly published name (https://lpsn.dsmz.de/genus/halalkalibacter, accessed on 5 February 2024) [14]. *Halalkalibacter* species have been isolated from various environments, such as soil, soda ponds, sea sediment, seawater, soda soil and saltpans [15,16,17,18,19,20,21]. Members of the genus *Halalkalibacter* were rod-shaped, endospore-forming, and Gram-staining positive or variable bacteria [13]. Most members of this genus were reported to be alkaliphilic, halotolerant or halophilic and aerobic, but some members were facultatively anaerobic [15,16,17,18,19,20,21,22]. They produce a variety of industrially valuable enzymes and were recognized as microorganisms of significant industrial importance [22]. In the present study, we describe the taxonomic position of strains HR 1-10^T^ and J-A-003^T^ and describe two new species.

## 2. Materials and Methods

### 2.1. Isolation and Preservation of Bacterial Strains

Strains HR 1-10^T^ and J-A-003^T^ were isolated from saline-alkali soil collected from Zhaodong County and Zhaozhou County in Heilongjiang, China, respectively. The samples were serially diluted and the suspension was spread on modified nutrient broth (mNB) supplemented with 5% (*w*/*v*) NaCl and 0.2% (*w*/*v*) Na_2_CO_3_, followed by incubation at 37 °C for 2 days. A single colony was picked and subsequently transferred and purified on APA agar medium [23] and maintained as glycerol suspensions (20%, *v*/*v*) at −80 °C and in lyophilized form in skimmed milk (15%, *w*/*v*) at 4 °C.

### 2.2. Morphological and Physiological Characteristics

Gram staining was performed using a Gram staining kit (Solarbio, Hangzhou, China) by following the manufacturer’s instructions. The shape of cells was observed by transmission electron microscopy (JEM-1400FLASH; JEOL; Tokyo, Japan). The motility of cells was identified by the development of turbidity in a tube containing a semi-solid APA medium. Endospores were examined according to the Schaeffer–Fulton staining method [24]. Growth at different temperatures (4, 15, 20, 29, 37, 45, 55 and 65 °C) was determined in liquid APA medium. The tolerance to salinity and alkalinity was determined in a liquid APA medium with various NaCl concentrations (0, 2, 5, 8, 10, 15 and 20%, *w*/*v*) and pH range (pH 6.0–12.0, at intervals of 1.0 pH unit). The pH of the basal medium was adjusted using the buffer system, as described by Narsing Rao et al. [25]. Catalase and oxidase activities were tested using 3% (*v*/*v*) H_2_O_2_ and the oxidase reagent, respectively [26]. Other enzyme activities, biochemical characteristics and utilization of carbon source were detected using API ZYM, API 20NE systems (bioMérieux, Grenoble, France) and GEN Ⅲ Microplate (Biolog, Newark, NJ, USA), by following the manufacturer’s instructions.

### 2.3. 16S rRNA Gene Sequencing and Phylogenetic Analysis

The genomic DNA was extracted using TIANamp genomic DNA kit (Tiangen Biotech, Beijing, China) according to the manufacturer’s instructions. PCR amplification was performed using primers, A 27F (5′-AGAGTTTGATCCTGGCTCAG-3′) and B 1492R (5′-CGGTTACCTTGTTACGACTT-3′). The PCR conditions were as follows: denaturation at 95 °C for 5 min, followed by 35 cycles of denaturation at 95 °C for 1 min, annealing at 56 °C for 1 min and extension at 72 °C for 2 min. At the end of the cycles, the reaction mixture was kept at 72 °C for 5 min and then cooled to 4 °C [23]. The PCR product was purified and cloned into the vector pMD19-T (Takara, Osaka, Japan) and sequenced. An almost full-length 16S rRNA gene sequence of strains HR 1-10^T^ and J-A-003^T^ was obtained and compared with type strains available in the EzBioCloud server (https://www.ezbiocloud.net/, accessed on 5 February 2024) [27]. The phylogenetic trees were constructed based on the 16S rRNA gene sequences of strains HR 1-10^T^ and J-A-003^T^ and related reference species. Multiple sequences were aligned in Molecular Evolutionary Genetics Analysis (MEGA X) software using the CLUSTAL_W algorithm [28]. Phylogenetic trees were constructed with neighbor-joining (NJ) [29], maximum parsimony (MP) [30] and maximum likelihood (ML) [31] methods using the MEGA X software [32]. Evolutionary distances were calculated using Kimura’s two-parameter model [33], and bootstrap values were calculated based on 1000 replications [34].

### 2.4. Chemotaxonomy

Chemotaxonomic characters of strains HR 1-10^T^ and J-A-003^T^ and the reference strains were observed using several standard methods under identical conditions. Analysis of the isomer of diaminopimelic acid was performed according to the procedures described by Rhuland et al. [35]. Menaquinones were extracted from freeze-dried biomass [36,37] and analyzed by HPLC [38,39]. Cellular fatty acid composition was analyzed according to the standard protocol of the Sherlock Microbial Identification System (Sherlock Version 6.1; MIDI database TSBA6; Newark, NJ, USA) [40]. Polar lipids were extracted as described by Minnikin et al. [41] and identified by two-dimensional thin-layer chromatography (TLC) [42].

### 2.5. Genome Sequencing and Comparison

The genomes of strains HR 1-10^T^ and J-A-003^T^ were sequenced by Majorbio Bio-pharm Technology Co., Ltd. (Shanghai, China) using an Illumina HiSeq X system. Reads of each data set were filtered, and high-quality paired-end reads were assembled using SOAPdenovo software (version 2.04) [43]. The genes were predicted using GeneMarkS [44]. The genomes were visualized using Proksee, with *Halalkalibacter krulwichiae* as a reference strain [45,46,47]. The prediction of tRNA genes was carried out using tRNAscan-SE (version 1.23) [48]. For analysis of genomic relatedness, the average nucleotide identity (ANI) value was calculated by using the pyANI with the blast method [49,50]. The digital DNA–DNA hybridization (dDDH) between genomes was calculated by the genome-to genome distance calculator (http://ggdc.dsmz.de/distcalc2.php, accessed on 5 February 2024) with BLAST+ and formula 2 [51,52,53]. Phylogenomic tree construction was performed using Anvi’o tool (version 7.1) [54,55]. Anvi-script-reformat-fasta was used to initially format the fasta file for the Anvi’o workflow. The scripts anvi-gen-contigs-database and anvi-run-hmms were executed to identify open reading frames and match genes in the contigs to single-copy core genes [56]. The genes present in HMM source ‘Bacteria_71’ [57] were taken and aligned using MUSCLE [58]. The resulting tree was visualized using MEGA X [32].

The prediction of protein-coding sequences (CDSs) was performed using Prodigal [59] with the “-p single” parameter. The predicted CDSs were annotated against the Kyoto Encyclopedia of Genes and Genomes (KEGG) databases using DIAMOND by applying *E*-values < 1 × 10^−5^ [60,61]. The functional annotation was performed with Rapid Annotations using Subsystems Technology (RAST) [62]. The secondary metabolism gene clusters were analyzed using antiSMASH (https://antismash.secondarymetabolites.org/, accessed on 5 February 2024) [63]. Pangenome analysis was performed by Roary [64] using annotated assemblies produced by Prokka [46].

### 2.6. Evaluation of Plant Growth Promotion

The nitrogen fixation ability of strains HR 1-10^T^ and J-A-003^T^ was tested using an Ashby nitrogen-free solid medium at 28 °C [65]. The 1-aminocyclopropane-1-carboxylate deaminase (ACCD) activity was determined on DF agar supplemented with ACC (ADF). The ADF medium was prepared according to the methods of Zhang et al. [66].

## 3. Results and Discussion

### 3.1. Morphological, Physiological, Biochemical and Phylogenetic Analysis

Strains HR 1-10^T^ and J-A-003^T^ were Gram-staining-positive, aerobic, motile and rod-shaped (0.3–0.8 µm wide and 1.5–4.6 µm long, Figure S1). Strain HR 1-10^T^ grew at 15–45 °C (optimum at 37 °C), pH 7.0–11.0 (optimum at pH 9.0) and could tolerate NaCl up to 15% (*w*/*v*), while strain J-A-003^T^ grew at 15–55 °C (optimum at 29 °C), pH 8.0–11.0 (optimum at pH 9.0) and could tolerate NaCl up to 10% (*w*/*v*). Strains HR 1-10^T^ and J-A-003^T^ were positive for esterase lipase (C8), leucine aryl-amidase and esculin ferric citrate hydrolysis but negative for lipase (C14), valine aryl-amidase, trypsin, *α*-chymotrypsin, *N*-acetyl-*β*-glucosaminidase, *α*-mannosidase, *α*-mannosidase, *β*-fucosidase, indole production, D-glucose fermentation, L-arginine di-hydrolase, gelatin hydrolysis, L-arabinose assimilation, D-mannose assimilation, D-mannitol assimilation, D-mannitol assimilation, *N*-acetyl-glucosamine assimilation, potassium gluconate assimilation, capric acid assimilation, adipic acid assimilation, adipic acid assimilation, trisodium citrate assimilation, and phenylacetic acid assimilation. Strain HR 1-10^T^, *H. wakoensis* JCM 9140^T^, *H. okhensis* JCM 10340^T^ and *H. kiskunsagensis* DSM 29791^T^ were positive for alkaline phosphatase. Strain HR 1-10^T^ was positive for urease but strain J-A-003^T^, *H. wakoensis* JCM 9140^T^, *H. okhensis* JCM 10340^T^, *H. kiskunsagensis* DSM 29791^T^ were negative. Strain HR 1-10^T^, *H. wakoensis* JCM 9140^T^, *H. okhensis* JCM 10340^T^ and *H. kiskunsagensis* DSM 29791^T^ were positive for *β*-galactosidase while J-A-003^T^ was negative. Both strains HR 1-10^T^ and J-A-003^T^ were negative for nitrate reduction. The results of *β*-galactosidase, *β*-glucuronidase, urease, D-glucose assimilation, D-maltose assimilation, and malic acid assimilation of strain HR 1-10^T^ were different from *H. wakoensis* JCM 9140^T^, *H. okhensis* JCM 10340^T^ (Table 1). Strain J-A-003^T^ can be differentiated from *H. kiskunsagensis* DSM 29791^T^ based on its distinctive biochemical profile, including characteristics such as alkaline phosphatase, cystine aryl-amidase, naphthol-AS-BI-phosphohydrolase, *α*-galactosidase, nitrate reduction, *β*-galactosidase, and malic acid assimilation. Detailed phenotypic, physiological, and biochemical characteristics of strains HR 1-10^T^, J-A-003^T^ and closely related species are mentioned in Table 1.

**Figure 1:**
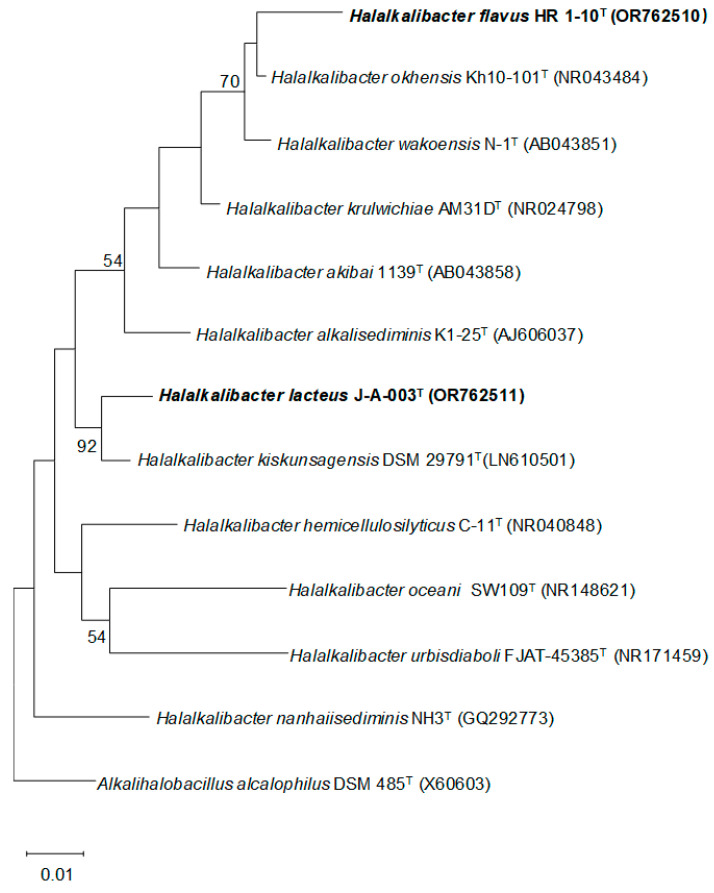
Maximum-likelihood phylogenetic tree based on 16S rRNA gene sequences of strains HR 1-10^T^, J-A-003^T^ and closest relatives. Bootstrap values (≥50%) based on 1000 replications are shown at branch nodes. *Alkalihalobacillus alcalophilus* DSM 485^T^ (X60603) is used as the outgroup. Bar, 0.01 represents substitutions per nucleotide position.

Strain HR 1-10^T^ shared the highest 16S rRNA gene sequence similarity with the type strain of *H. wakoensis* (98.3%) while strain J-A-003^T^ shared the highest 16S rRNA gene sequence similarity with the type strain of *H. kiskunsagensis* (98.6%). In the ML tree (Figure 1), strain HR 1-10^T^ clade with *H. okhensis* JCM 10340^T^ and *H. wakoensis* JCM 9140^T^, while J-A-003^T^ clade with *H. kiskunsagensis* DSM 29791^T^. The clade was stable when the trees were re-constructed using MP and NJ methods (Figures S2 and S3). The above results preliminarily indicated that strains HR 1-10^T^ and J-A-003^T^ should be affiliated to the genus *Halalkalibacter*.

### 3.2. Chemotaxonomic Characterization

Strains HR 1-10^T^ and J-A-003^T^ consist of *meso*-2,6-diaminopimelic acid as cell-wall diamino acid. Strain J-A-003^T^ consists of MK-7 as menaquinone, while HR 1-10^T^ consists of MK-6 and MK-7. The results of cell-wall diamino acid and menaquinone of strains were similar to the species of the genus of *Halalkalibacter* [9,10,14,15]. The major fatty acids (>10%) in both HR 1-10^T^ and J-A-003^T^ were C_16:0_ and anteiso-C_15:0_. In addition, iso-C_15:0_ was >10% in strain J-A-003^T^. The major fatty acids profile of strains HR 1-10^T^ and J-A-003^T^ were similar to other closest relatives but their portions varied (Table 2). Strains HR 1-10^T^ and J-A-003^T^ exhibited similar polar lipid profiles, including diphosphatidyl-glycerol (DPG), phosphatidylglycerol (PG), and an unidentified phospholipid (PL). However, strain J-A-003^T^ additionally contained phosphatidylethanolamine (PE), while strain HR 1-10^T^ additionally contained an unidentified amino-phospholipid (APL) (Figure S4).

### 3.3. Genome Analysis

**Figure 2:**
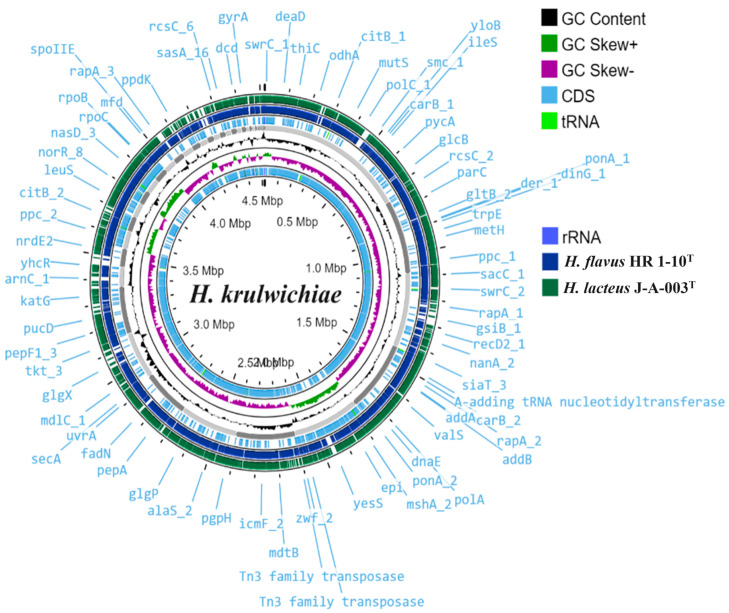
Genome comparison by Proksee using *Halalkalibacter krulwichiae* as a reference genome.

**Figure 3:**
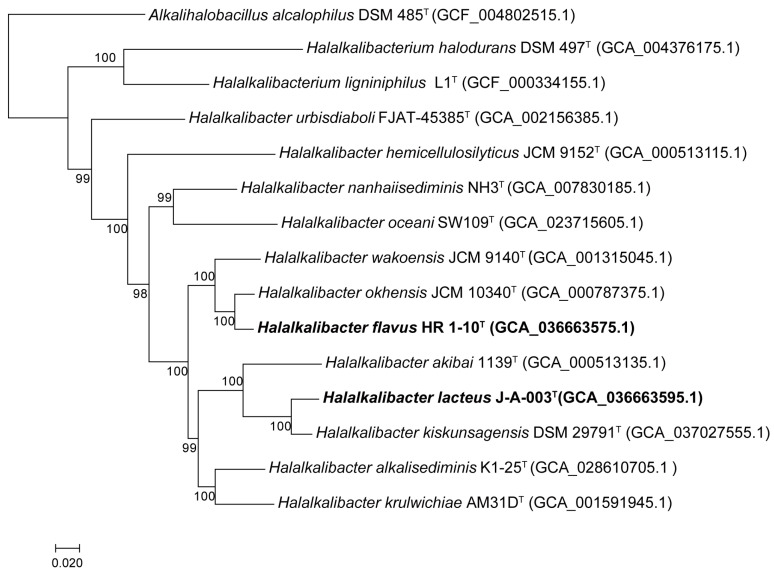
Phylogenomic tree based on 71 bacterial single-copy genes showing the relationship of strains HR 1-10^T^, J-A-003^T^ and members of the genus *Halalkalibacter*. Bootstrap values (expressed as percentages of 1000 replications) greater than 50% are shown at branch points. Bar, 0.02 represents substitution per nucleotide position. *Alkalihalobacillus alcalophilus* DSM 485^T^ (GCA_004802515.1) is used as the outgroup.

The genome size of strains HR 1-10^T^ and J-A-003^T^ were 5,127,176 bp and 4,471,997 bp, respectively. Genome visualization and comparison of strains HR 1-10^T^ and J-A-003^T^ are depicted in Figure 2. The genomic DNA G+C contents of strains HR 1-10^T^ and J-A-003^T^ were 38.3% and 38.4%, respectively. In the phylogenomic tree, strains HR 1-10^T^ and J-A-003^T^ clustered with the members of the genus *Halalkalibacter* (Figure 3). A total of 2790 and 2401 genes were assigned as putative functions, while the remaining protein-coding genes were annotated as hypothetical proteins in strains HR 1-10^T^ and J-A-003^T^, respectively.

Strains HR 1-10^T^ and J-A-003^T^ encode genes for glycolysis, citric cycle and pentose phosphate pathway. Strains HR 1-10^T^ and J-A-003^T^ also encode genes for nitrate assimilation and assimilatory sulfate reduction. Strains HR 1-10^T^ and J-A-003^T^ demonstrated tolerance to high salt concentrations. Consequently, a genome analysis was conducted to evaluate the mechanism underlying this trait. Microorganisms overcome salt stress by salt-in (by accumulation of high concentrations of inorganic salts or ions in their cytosol) and salt-out strategies (accumulation of high concentrations of compatible solutes) [67]. In the present study, strains HR 1-10^T^ and J-A-003^T^ encode genes for Na^+^/H^+^ antiporter, K^+^ transport protein and glycogen, ectoine, proline biosynthesis and betaine biosynthesis to overcome salt stress. In addition, both strains also encode genes for ornithine, lysine and arginine biosynthesis. Functional annotation using RAST showed that strains HR 1-10^T^ and J-A-003^T^ encode most genes linked to amino acids and derivatives, protein and carbohydrate metabolism (Figure S5). Genes related to cofactors and vitamins were also observed. The metabolism distributions for genes against the KEGG database are mentioned in Table S1.

The antiSMASH server also predicted ectoine biosynthesis gene clusters in strains HR 1-10^T^ and J-A-003^T^, showing more than 50% similarities to known biosynthetic gene clusters. In addition, strain HR 1-10^T^ showed the presence of koranmine biosynthesis gene clusters with 87% similarities to known biosynthetic gene clusters.

**Figure 4:**
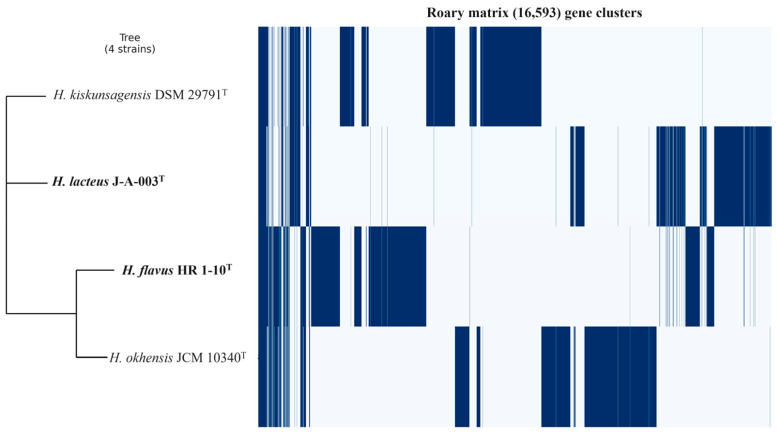
Pangenome analysis (Roary matrix) showing the presence and absence of gene clusters.

The ANI and dDDH values between strain J-A-003^T^ and *H. kiskunsagensis* DSM 29791^T^ were 86.5% and 36.7%, respectively. The ANI and dDDH values between strain HR 1-10^T^ and *H. okhensis* JCM 10340^T^ were 84.3% and 30.9%, respectively, and those relative to *H. wakoensis* JCM 9140^T^ were 78.4% and 19.8%, respectively. The ANI (95–96%) and dDDH (70%) values were lower than the threshold value recommended for distinguishing novel species [68,69,70]. Pangenome analysis detected 16,593 gene clusters (Figure 4). The pangenome analysis showed different gene clusters among the present study strains and with other *Halalkalibacter* species.

### 3.4. Plant Growth Promotion Abilities

Numerous halophilic and halotolerant microorganisms have been found to have characteristics that encourage plant growth [71]. They apply different strategies to elevate plant growth, for instance, through nitrogen fixation and ACC deaminase activity. Biological nitrogen fixation is a microbial process that utilizes nitrogenase to convert atmospheric nitrogen into ammonium, a form easily absorbable by roots, thereby facilitating plant growth [72]. Aminocyclopropane-1-carboxylic acid (ACC) serves as a precursor to ethylene, with its levels increasing in plants facing biotic and abiotic stress [73]. Various soil microorganisms have been identified as producers of ACC deaminase (ACCd), an enzyme that breaks down ACC, consequently decreasing stress-induced ethylene levels in host plants. These microorganisms possess the capability to hydrolyze ACC into α-keto-butyrate and ammonia, potentially reducing ethylene levels in plants and easing growth inhibition triggered by excessive ethylene [74]. Strains HR 1-10^T^ and J-A-003^T^ grew well on Ashby nitrogen-free solid medium and ADF medium, implying that the strains harbor nitrogenase and 1-aminocyclopropane-1-carboxylate deaminase (ACCD) activity (Figure S6). The results indicate that the strains HR 1-10^T^ and J-A-003^T^ could be potential candidates as plant growth promoters.

## 4. Conclusions

Two strains HR 1-10^T^ and J-A-003^T^ were isolated from saline-alkali soil. 16S rRNA gene sequence and phylogenetic analysis suggest that these strains were members of the genus *Halalkalibacter.* They demonstrated tolerance to high salt concentrations and also produce enzymes for plant growth promotion.

The morphological, biochemical, menaquinone, fatty acids and polar lipids profiles differentiate strains HR 1-10^T^ and J-A-003^T^ from each other, as well as with other species of *Halalkalibacter.* The genome relatedness value between HR 1-10^T^ and J-A-003^T^ and with *Halalkalibacter* species were below the cut-off value for species delineation. Based upon the results, strains HR 1-10^T^ and J-A-003^T^ represent two novel species of the genus *Halalkalibacter* and their names were proposed based on the colony color. The name *Halalkalibacter flavus* sp. nov., is proposed for HR 1-10^T^ (=GDMCC 1.2946^T^ = MCCC 1K08312^T^ = JCM 36285^T^), while *Halalkalibacter lacteus* sp. nov., proposed for J-A-003^T^ (=GDMCC 1.2949^T^ = MCCC 1K08417^T^ = JCM 36286^T^).

Description of *Halalkalibacter flavus* sp. nov.

*Halalkalibacter flavus* (*fla’vus.* L. masc. adj. *flavus*, yellow).

Cells are Gram-staining-positive, aerobic, motile and rod-shaped (0.6–0.8 µm wide and 1.5–1.7 µm long). Colonies are yellow. Growth occurs at pH 7.0–11.0 (optimum pH 9.0), 15–45 °C (optimum at 37 °C) and can tolerate NaCl up to 15% (*w*/*v*). Catalase, amylase and cellulase are positive, but gelatin and oxidase are negative. Hydrolysis of Tweens 20, 40, 60 and 80 are positive. Nitrate reduction, coagulation and peptization of milk, indole and H_2_S production are negative. In the Biolog GEN III Microplate system, the following substrates are used as sole carbon sources: glucuronamide, D-galactose, D-galacturonic acid, L-galactonic acid lactone, L-rhamnose and D-glucuronic acid. The API 20NE test showed positive for urease, D-glucose hydrolysis, aesculin hydrolysis, malic acid assimilation and galactosidase, but negative for arginine di-hydrolase, gelatin hydrolysis, nitrate reduction, indole production, glucose fermentation and assimilation of L-arabinose, D-mannose, D-mannitol, *N*-acetyl glucosamine, D-maltose, potassium gluconate, capric acid, adipic acid, trisodium citrate and phenylacetic acid. The API ZYM test showed positive for alkaline phosphatase, esterase (C4), esterase lipase (C8), leucine aryl-amidase, naphthol-AS-BI-phosphohydrolase, *β*-galactosidase, *α*-glucosidase and *β*-glucosidase, but negative for lipase (C14), valine aryl-amidase, cystine aryl-amidase, trypsin, α-chymotrypsin, acid phosphatase, α-galactosidase, *N*-acetyl-β-glucosaminidase, *α*-mannosidase and *β*-fucosidase. The menaquinone is MK-6 and MK-7, and *meso*-2,6-diaminopimelic acid as the cell-wall diamino acid. The major fatty acids are anteiso-C_15:0_ and C_16:0_. The polar lipids are diphosphatidyl-glycerol, phosphatidylglycerol, an unidentified amino-phospholipids and unidentified phospholipid. The genomic DNA G+C content is 38.3%.

The type strain HR 1-10^T^ (=GDMCC 1.2946^T^ = MCCC 1K08312^T^ = JCM 36285^T^) was isolated from soil in Heilongjiang province, China. The GenBank/EMBL/DDBJ accession numbers for the 16S rRNA gene and genome sequence are OR762510 and JAWRVK000000000, respectively.

Description of *Halalkalibacter lacteus* sp. nov.

*Halalkalibacter lacteus* (*lac’te.us.* L. masc. adj. *lacteus*, milky-white).

Cells are Gram-staining-positive, aerobic, motile and rod-shaped (0.6–0.8 µm wide and 1.8–4.6 µm long). Colonies are milky-white. Growth occurs at pH 8.0–11.0 (optimum pH 9.0), 15–55 °C (optimum at 29 °C) and can tolerate NaCl up to 10% (*w*/*v*). Catalase, amylase, oxidase and cellulase are positive, but gelatin is negative. Hydrolysis of Tweens 20, 40, 60 and 80, nitrate reduction, coagulation and peptization of milk, indole and H_2_S production are negative. In the Biolog GEN III Microplate system, the following substrates are used as sole carbon sources: glucuronamide, L-fructose, 3-methyl glucose, D-fructose, L-fructose, L-rhamnose, D-fructose-6-PO_4_ and D-glucuronic acid. The API 20NE test showed positive for aesculin hydrolysis and malic acid assimilation, but negative for urease, D-glucose hydrolysis, arginine di-hydrolase, gelatin hydrolysis, nitrate reduction, indole production, galactosidase, glucose fermentation and assimilation of L-arabinose, D-mannose, D-mannitol, *N*-acetyl glucosamine, D-maltose, potassium gluconate, capric acid, adipic acid, trisodium citrate and phenylacetic acid. The API ZYM test showed positive for esterase (C4), esterase lipase (C8), leucine aryl-amidase, naphthol-AS-BI-phosphohydrolase, *α*-glucosidase and *β*-glucosidase, but negative for alkaline phosphatase, lipase (C14), valine aryl-amidase, cystine aryl-amidase, trypsin, *α*-chymotrypsin, acid phosphatase, *α*-galactosidase, *β*-galactosidase, *N*-acetyl-*β*-glucosaminidase, *α*-mannosidase and *β*-fucosidase. The menaquinone is MK-7 and *meso*-2,6-diaminopimelic acid is the cell-wall diamino acid. The major fatty acids are anteiso-C_15:0_, iso-C_15:0_ and C_16:0_. The polar lipids are diphosphatidyl-glycerol, phosphatidylglycerol, phosphatidylethanolamine and an unidentified phospholipid. The genomic DNA G+C content is 38.4%.

The type strain J-A-003^T^ (=GDMCC 1.2949^T^ = MCCC 1K08417^T^ = JCM 36286^T^) was isolated from soil in Heilongjiang province, China. The GenBank/EMBL/DDBJ accession numbers for the 16S rRNA gene and genome sequence are OR762511 and JAWRVL000000000, respectively.

## Figures and Tables

**Table 1:** Differential characteristics of strains HR 1-10^T^, J-A-003^T^ and closest relatives. Taxa: 1. strain HR 1-10^T^; 2. strain J-A-003^T^; 3. *H. wakoensis* JCM 9140^T^; 4. *H. okhensis* JCM 10340^T^; 5. *H. kiskunsagensis* DSM 29791^T^. All data are from this study. Note: +, positive; −, negative.

Characteristic	1	2	3	4	5
Temperature range (°C) for growth	15–45	4–55	20–45	20–45	4–37
pH range for growth	7.0–11.0	8.0–11.0	7.0–10.0	7.0–10.0	8.0–12.0
NaCl tolerance (%, *w*/*v*) for growth	0–15	0–10	0–15	0–15	0–15
API ZYM test					
Alkaline phosphatase	+	−	+	+	+
Esterase (C4)	+	+	−	+	+
Cystine arylamidase	−	−	−	−	+
Acid phosphatase	−	+	+	−	+
Naphthol-AS-BI-phosphohydrolase	+	+	+	+	−
*α*-Galactosidase	−	−	−	−	+
*β*-Galactosidase	+	−	−	−	−
*β*-Glucuronidase	+	−	−	−	−
*β*-Glucosidase	+	+	+	−	+
API 20NE test					
Nitrate reduction	−	−	−	+	+
Urease	+	−	−	−	−
*β*-galactosidase	+	−	+	+	+
D-Glucose assimilation	+	−	−	−	−
D-Maltose assimilation	+	−	−	−	−
Malic acid assimilation	+	+	−	−	−

**Table 2:** Cellular fatty acid profiles of strain HR 1-10^T^, J-A-003^T^ and their closest relatives.

Fatty Acids (%)	1	2	3	4	5
**Saturated:**					
C_12:0_	1.48	0.9	2.0	1.3	1.4
C_14:0_	2.06	4.4	1.8	1.1	3.0
C_16:0_	**29.3**	**17.8**	**25.3**	**12.8**	**16.6**
C_17:0_	1.1	tr	0.8	tr	0.66
C_18:0_	1.6	0.8	tr	tr	1.3
**Unsaturated:**					
C_16:1_*ω*11*c*	0.5	4.2	1.6	0.5	tr
C_18:3_*ω*6*c*	1.5	tr	tr	1.5	0.7
**Branched:**					
iso-C_10:0_	0.7	tr	tr	tr	tr
iso-C_13:0_	tr	0.6	tr	tr	0.6
iso-C_14:0_	4.4	5.5	0.6	3.7	4.8
anteiso-C_14:0_	3.3	1.5	2.6	tr	1.9
iso-C_15:0_	7.5	**17.8**	8.1	8.6	**13.1**
anteiso-C_15:0_	**27.1**	**32.7**	**33.2**	**47.1**	**33.1**
iso-C_16:0_	3.9	2.3	3.1	4.8	3.6
iso-C_17:0_	2.7	2.3	3.9	3.0	3.0
anteiso-C_17:0_	3.4	3.2	6.7	8.6	3.3
Summed features *					
3	4.6	1.9	2.5	2.0	7.8
9	1.2	tr	1.6	1.8	tr

Taxa: 1. strain HR 1-10^T^; 2. strain J-A-003^T^; 3. *H. wakoensis* JCM 9140^T^; 4. *H. okhensis* JCM 10340^T^; 5. *H. kiskunsagensis* DSM 29791^T^. All data were obtained in this study. Values are percentages of the total fatty acids. Note: tr, fatty acids less than 0.5%. Fatty acids that represented >10% are highlighted in bold. * Summed Features are fatty acids that cannot be resolved reliably from another fatty acid using the chromatographic conditions chosen. The MIDI system groups these fatty acids together as one feature with a single percentage of the total. Summed feature 3 (C_16 : 1_*ω*7*c* and/or C_16 : 1_*ω*6*c*) and summed feature 9 (iso-_C17:1_*ω*9*c* and/or C_16:0_ 10-methyl).

## Data Availability

All data generated or analyzed during this study are included.

## Supplementary Materials

The following supporting information can be downloaded at: https://www.mdpi.com/article/10.3390/microorganisms12050950/s1, Table S1: Number of genes associated with the metabolism of strains HR 1-10^T^, J-A-003^T^ and closest relatives. Taxa: 1. strain HR 1-10^T^; 2. strain J-A-003^T^; 3. *H. wakoensis* JCM 9140^T^; 4. *H. okhensis* JCM 10340^T^; 5. *H. kiskunsagensis* DSM 29791^T^; Figure S1: Transmission electronic micrograph of strains: (a) HR 1-10^T^ and (b) J-A-003^T^ grown on APA agar at 37 ºC for 3 days. Bars, 1 μm; Figure S2: Maximum-parsimony phylogenetic tree based on 16S rRNA gene sequences of HR 1-10^T^, J-A-003^T^ and closest relatives. Bootstrap values (≥50%) based on 1000 replications are shown at branch nodes. *Alkalihalobacillus alcalophilus* DSM 485^T^ (X60603) is used as the outgroup. Bar, 10 represents substitutions per nucleotide position; Figure S3: Neighbor-joining phylogenetic tree based on 16S rRNA gene sequences of strains HR 1-10^T^, J-A-003^T^ and other closest relatives. Bootstrap values (≥50%) based on 1000 replications are shown at branch nodes. *Alkalihalobacillus alcalophilus* DSM 485^T^ (X60603) is used as the outgroup. Bar, 0.005 represents substitutions per nucleotide position; Figure S4: Two-dimensional TLC patterns of the total polar lipids from strains (a), HR 1-10^T^ and (b) J-A-003^T^, the plates were developed using chloroform/methanol/water (65:25:4 *v*/*v*) as the first direction, followed by chloroform/acetic acid/methanol/water (80:15:12:4 *v*/*v*) as the second direction; Figure S5: Functional annotation using RAST for strains HR 1-10^T^ (a) and J-A-003T (b); Figure S6: Evaluation of plant growth promotion. Abbreviations: DPG, diphosphatidylglycerol; PG, phosphatidylglycerol; APL, unidentified aminophospholipids; PL, unidentified phospholipid; PE, Phosphatidylethanolamine.
