# Supplementary material for: Two Novel Alkaliphilic Species Isolated from Saline-Alkali Soil in China: Halalkalibacter flavus sp. nov., and Halalkalibacter lacteus sp. nov
Source: Microorganisms. 2024 May 8;12(5):950. doi: 10.3390/microorganisms12050950 (PMC11123755; doi:10.3390/microorganisms12050950)

Supplementary materials

Two novel alkaliphilic species isolated from saline-alkali soil in China: *Halalkalibacter flavus* sp. nov. and *Halalkalibacter lacteus* sp. nov

Pin-Jiao Jin[1], Lei Sun[1], Yong-Hong Liu[2], Kang-Kang Wang[1], Manik Prabhu Narsing Rao[5], Osama Abdalla Abdelshafy Mohamad[2], Bao-Zhu Fang[2,3], Li Li[2], Lei Gao[2], Wen-Jun Li[2,4], Shuang Wang[1,2,*]

[1]Heilongjiang Academy of Black Soil Conservation & Utilization, Postdoctoral Station of Heilongjiang Academy of Agricultural Sciences, Harbin 150086, PR China

[2]State Key Laboratory of Desert and Oasis Ecology, Key Laboratory of Ecological Safety and Sustainable Development in Arid Lands, Xinjiang Institute of Ecology and Geography, Chinese Academy of Sciences, Urumqi, 830011, PR China

[3]Xinjiang Key Laboratory of Biodiversity Conservation and Application in Arid Lands, Xinjiang Institute of Ecology and Geography, Chinese Academy of Sciences, Urumqi, 830011, PR China

[4]State Key Laboratory of Biocontrol and Guangdong Key Laboratory of Plant Resources, School of Life Sciences, Sun Yat-Sen University, Guangzhou 510275, PR China

[5]Instituto de Ciencias Aplicadas, Facultad de Ingeniería, Universidad Autónoma de Chile, Sede Talca, Talca 3460000, Chile

***Authors for correspondence:**

Shuang Wang, Heilongjiang Academy of Black Soil Conservation & Utilization, 368 Xuefu Road, Harbin 150086, PR China, Tel & Fax: +86 0451-86668726, E-mail: wangshuang0726@163.com

Running title: *Halalkalibacter flavus* sp. nov., and *Halalkalibacter lacteus* sp. nov.

Table S1. Number of genes associated with metabolism of strains HR1-10^T, J-A-003^T and other closest relatives. Taxa: 1. strain HR1-10^T; 2. strain J-A-003^T; 3. *H. wakoensis* JCM 9140^T; 4. *H. okhensis* JCM 10340^T; 5. *H. kiskunsagensis* DSM 29791^T.

**Metabolism**	**1**	**2**	**3**	**4**	**5**
Amino acid metabolism	380	302	399	350	325
Biosynthesis of other secondary metabolites	41	33	36	31	36
Carbohydrate metabolism	557	449	453	489	337
Cell growth and death	13	17	16	13	30
Cell motility	34	19	97	81	40
Energy metabolism	182	177	172	163	182
Folding, sorting and degradation	56	55	56	50	42
Glycan biosynthesis and metabolism	33	32	32	32	32
Lipid metabolism	107	80	130	102	82
Membrane transport	215	157	130	165	186
Metabolism of cofactors and vitamins	176	172	162	155	144
Metabolism of other amino acids	74	65	62	64	80
Metabolism of terpenoids and polyketides	38	33	52	33	43
Nucleotide metabolism	120	133	136	126	112
Overview	364	336	375	355	314
Replication and repair	83	96	103	89	65
Signal transduction	125	95	144	133	90
Transcription	5	5	6	5	4
Translation	90	89	100	90	74
Transport and catabolism	11	11	12	14	11
Xenobiotics biodegradation and metabolism	86	45	111	79	129

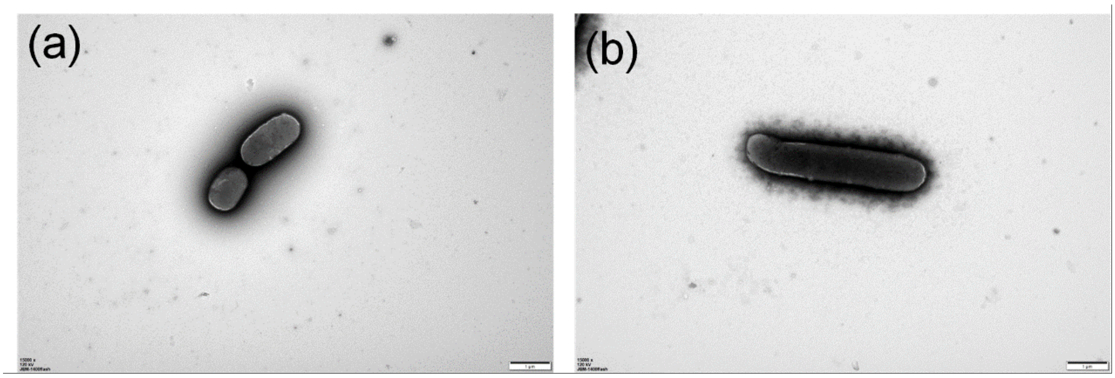

Figure S1. Transmission electronic micrograph of strains: a) HR1-10^{T} and b) J-A-003^{T} grown on APA agar at 37 ºC for 3 days. Bars, 1 μm.

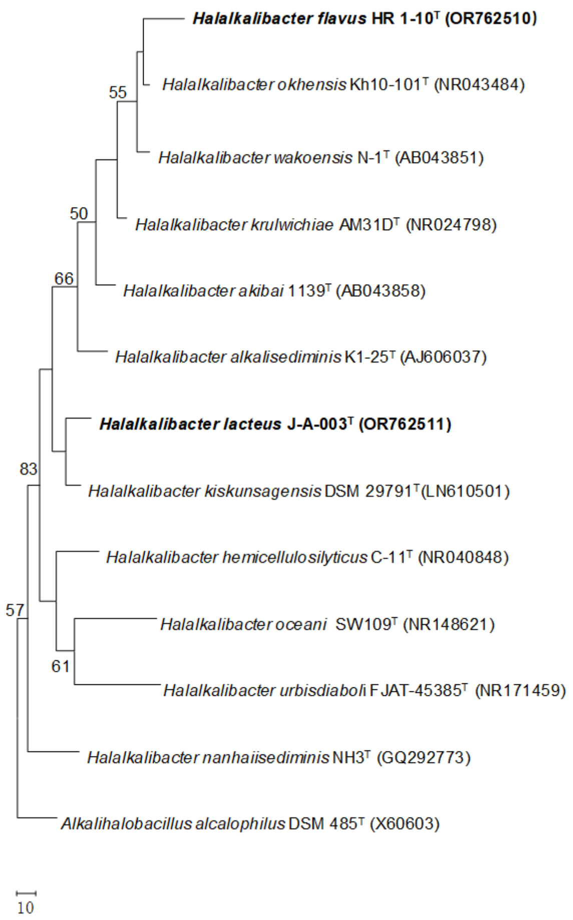

Figure S2 Maximum-parsimony phylogenetic tree based on 16S rRNA gene sequences of HR1-10^T, J-A-003^T and other closest relatives. Bootstrap values (≥50 %) based on 1000 replications are shown at branch nodes. *Alkalihalobacillus alcalophilus* DSM 485^T (X60603) is used as the outgroup. Bar, 10 represents substitutions per nucleotide position.

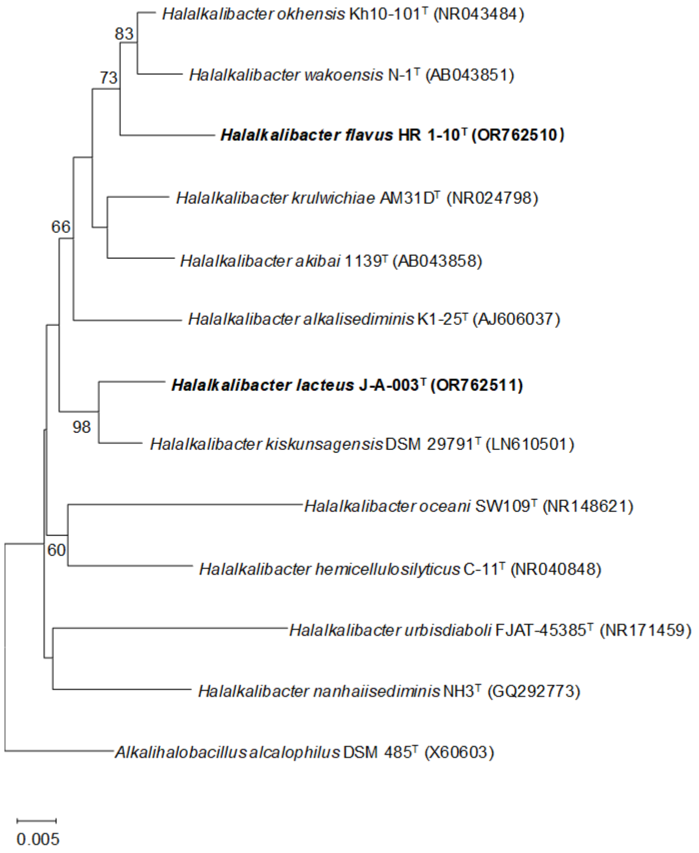

Figure S3. Neighbor-joining phylogenetic tree based on 16S rRNA gene sequences of strains HR1-10^T, J-A-003^T and other closest relatives. Bootstrap values (≥50%) based on 1000 replications are shown at branch nodes. *Alkalihalobacillus alcalophilus* DSM 485^T (X60603) is used as the outgroup. Bar, 0.005 represents substitutions per nucleotide position.

Figure S4 Two-dimensional TLC patterns of the total polar lipids from strains (a), HR1-10[T] and (b) J-A-003[T], the plates were developed using chloroform/methanol/water (65:25:4 v/v) as the first direction, followed by chloroform/acetic acid/methanol/water (80:15:12:4 v/v) as the second direction.

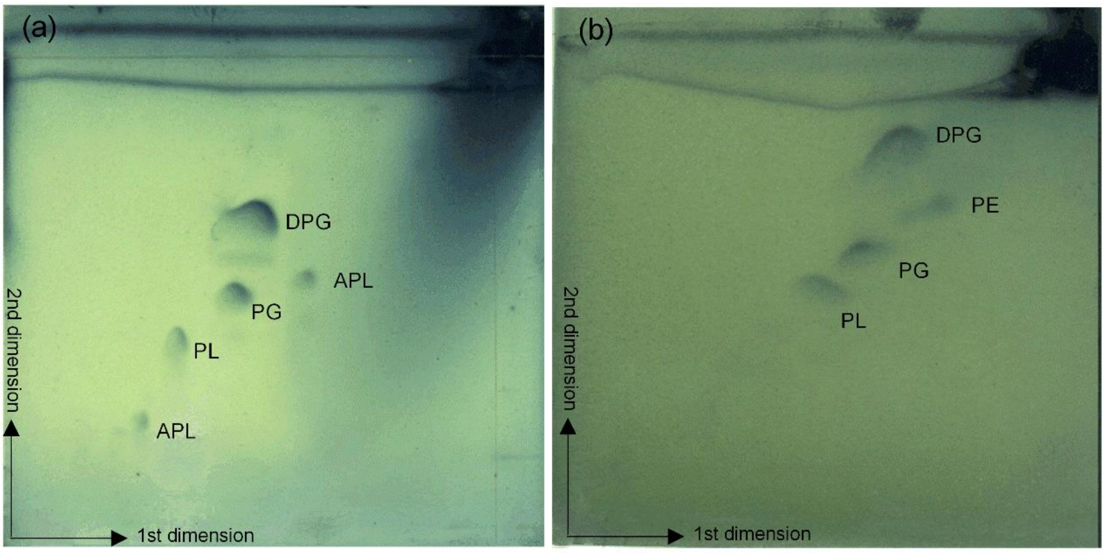

Abbreviations: DPG, diphosphatidylglycerol; PG, phosphatidylglycerol; APL, unidentified aminophospholipids; PL, unidentified phospholipid; PE, Phosphatidylethanolamine.

Figure S5 Functional annotation using RAST for strains HR1-10^T (a) and J-A-003^T (b).

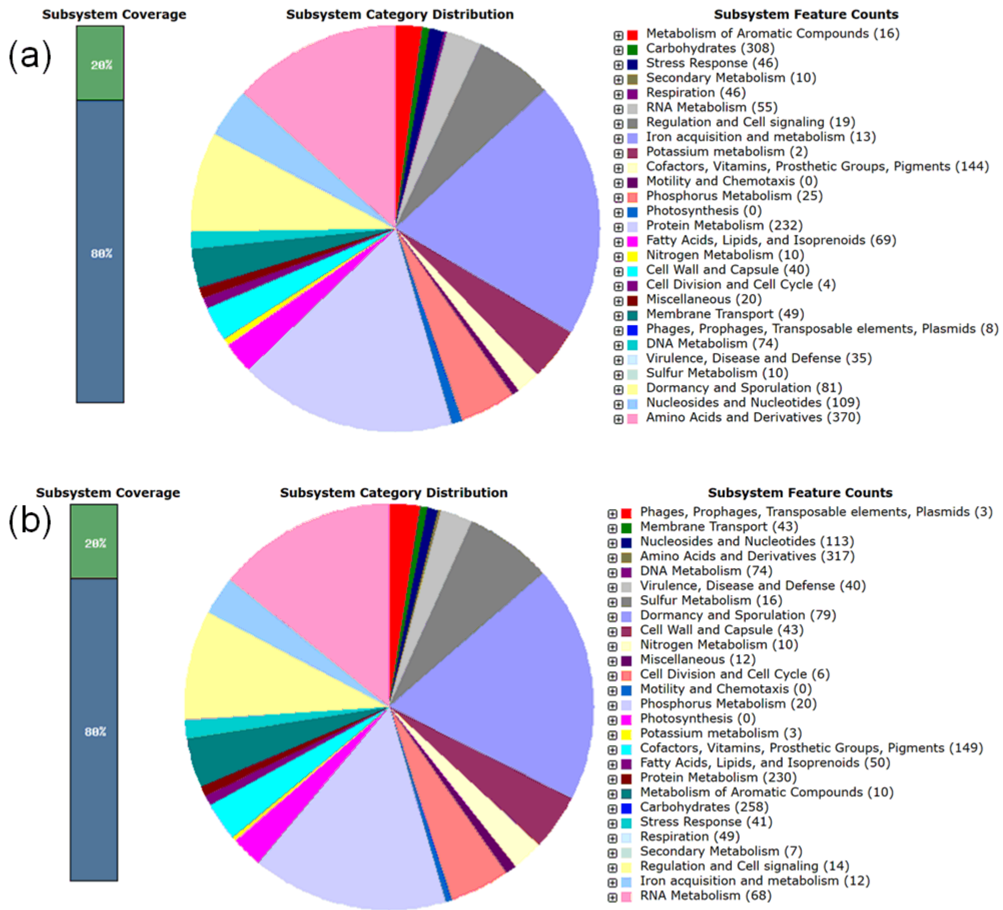

Figure S6 Evaluation of plant growth promotion.

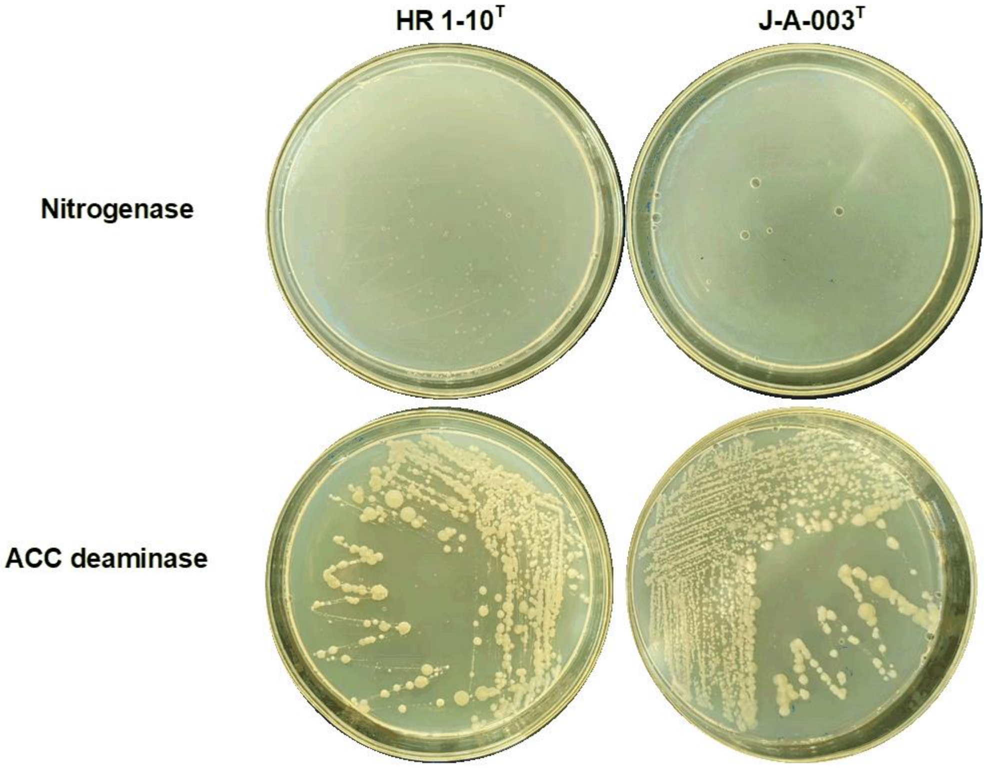

Supplement: Supplementary file 1 [file microorganisms-12-00950-s001.zip › microorganisms-2923564-supplementary.pdf]
